# Long non-coding RNA *AGAP2-AS1* increases the invasiveness of papillary thyroid cancer

**DOI:** 10.18632/aging.103570

**Published:** 2020-09-18

**Authors:** Liang Shao, Wei Sun, Hao Zhang, Ping Zhang, Zhihong Wang, Wenwu Dong, Liang He, Ting Zhang, Yuan Qin

**Affiliations:** 1 Department of Thyroid Surgery, The First Hospital of China Medical University, Shenyang 110001, Liaoning Province, P. R. China

**Keywords:** LncRNA, AGAP2-AS1, papillary thyroid cancer, miR-424-5p, MMP2

## Abstract

Papillary thyroid cancer (PTC) is considered a low hazard endocrine system cancer, but a considerable number of patients have poor prognosis because of lymph node metastasis and invasion of surrounding tissues. In this study, we analyzed the expression and function of the long non-coding RNA (lncRNA) *AGAP2-AS1* in PTC. We found that *AGAP2-AS1* expression was significantly higher in human PTC tissues than adjacent noncancerous tissues (n=110; p<0.01) and correlated with lymph node metastasis (p=0.01) and tumor-node-metastasis stage (p=0.006). *AGAP2-AS1* downregulation decreased migration and invasion by PTC cells, and reduced expression of matrix metalloproteinase-2 (MMP2). *AGAP2-AS*1 upregulated MMP2 expression by competitively binding to microRNA-425-5p. In addition, miR-424-5p expression was decreased in PTC tissues and correlates negatively with the *AGAP2-AS1* levels. These results demonstrate that *AGAP2-AS1* expression is significantly elevated in PTC tissues and that, by binding to miRNA-425-5p, it upregulates the MMP2 expression, thereby increasing the invasiveness and migration capacity of PTC cells.

## INTRODUCTION

Papillary thyroid cancer (PTC) is one the most common malignant tumors of the endocrine system [1, 2]. Despite a good prognosis in most PTC patients, some patients have unsatisfactory outcomes because of tumor persistence, and have a recurrence rate of approximatively 30% [3–5]. The 10-year survival rate of patients who develop distant metastases is less than 40% [6]. Therefore, it is crucial to find new molecular markers to improve the initial diagnosis, which could reveal the mechanisms of PTC tumorigenesis, and improve patient prognosis.

Deregulation of genes associated with MAPK and PI3K/AKT serine/threonine kinase signaling pathways accounts for 90–95% of thyroid cancer cases [7, 8]. Long non-coding RNAs (lncRNAs) are RNA molecules of over 200 nucleotides; they are located in the cell nucleus and cytoplasm [9]. LncRNAs possess limited or no protein-coding capacity, and function as regulators in cancer progression [10–12]. They exert their functions by regulating chromatin accessibility and gene expression. For example, in hepatocellular carcinoma, *lncAKHE* promotes cell growth and migration through NOTCH-2 signaling [13]. In PTC, the lncRNA *NEAT1_*2 inhibits apoptosis and promotes cell migration, growth, and invasion by acting as a competing endogenous RNA (ceRNA) to regulate the expression of *ATAD2* by sponging miR-106b-5p [14]. Thousands of lncRNAs are dysregulated in thyroid cancer; however, the function of most of them in cancer development is unknown.

The lncRNA AGAP2 antisense RNA 1 (*AGAP2-AS1*) is a 1567 nt antisense lncRNA transcribed from a gene located at 12q14.1. *AGAP2-AS1* plays important roles in hepatocellular carcinoma, pancreatic cancer, and gastric cancer [15–17]; however, its role and mechanism of function in PTC are not known. Matrix metalloproteinases (MMPs) comprise a family of zinc-dependent endoproteases that have important roles in tissue remodeling and degradation of proteins of the extracellular matrix (ECM) [18, 19].

The aim of this study was to determine the role of *AGAP2-AS1* in PTC, and to investigate whether it might provide a new target for PTC diagnosis and treatment. Our results demonstrate that the expression of *AGAP2-AS1* is increased in PTC tissues, and by binding to miR-424-5p, it upregulates the MMP2 expression, resulting in increased migration and invasion of PTC cells. These findings indicate that *AGAP2-AS1* might serve as a novel target for diagnosis and treatment of PTC.

## RESULTS

### *AGAP2-AS1* expression is increased in PTC tissues and correlates with TNM stage and lymph node metastasis in PTC

**Figure 1:**
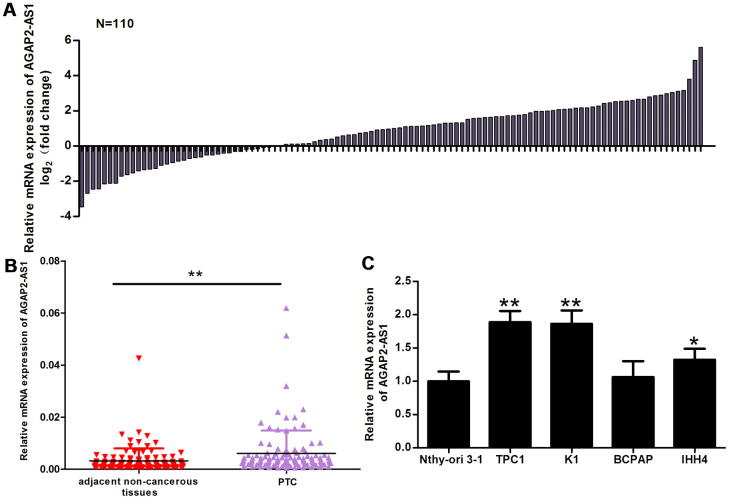
***AGAP2-AS1* expression is increased in PTC tissues and cells.** (**A**) The fold change of *AGAP2-AS1* expression between PTC tissues and paired non-cancerous tissues. (**B**) RT-PCR of *AGAP2-AS1* levels in 110 pairs of PTC tissues and paired non-cancerous tissues. The Wilcoxon signed-rank test was used to analyze the differences between the two groups; data are presented as the median with a range. **P < 0.01. (**C**) RT-PRC of *AGAP2-AS1* levels in TPC1, K1, BCPAP, and IHH4 PTC cells, and in a normal human thyroid follicular epithelial cell line (Nthy-ori 3-1). Data were analyzed using an independent samples t-test. *P < 0.05, **P < 0.01 *vs*. the Nthy-ori 3-1 group.

Using qRT-PCR, we analyzed *AGAP2-AS1* expression in 110 paired PTC tissues and adjacent non-cancerous tissues. Compared with non-cancerous tissues, the *AGAP2-AS1* expression was significantly increased in PTC tissues (Figure 1A, 1B). The median value of *AGAP2-AS1* expression in PTC tissues was 0.6553. Using this median, we divided the 110 patients into two groups: Those with a high relative expression of *AGAP2-AS1* (≥ 0.6553; n = 56) and those with a low relative expression of *AGAP2-AS1* (< 0.6553; n = 54). The results in Table 1 show that the relative expression of *AGAP2-AS1* was associated with the TNM stage (p = 0.006) and lymph node metastasis (p = 0.01). There was no significant correlation between the two groups and other clinicopathological characteristics.

**Table 1:** Correlation between AGAP2-AS1 expression and clinicopathological features in papillary thyroid cancer (PTC) (n = 110).

**Characteristics**	**n**	**High expression(%)**	**Low expression(%)**	***p***
Gender				
Male	37	18(48.65)	19(51.35)	0.84
Female	73	37(50.68)	36(49.32)	
Age(years)				
<45	80	41(51.25)	39(48.75)	0.669
≥45	30	14(46.67)	16(53.33)	
Extrathyroidal extension		
Yes	40	21(52.5)	19(47.5)	0.692
No	70	34(48.57)	36(51.43)	
TNM staging				
I-II	68	27(39.71)	41(60.29)	0.006
III-IV	42	28(66.67)	14(33.33)	
Lymph node metastasis		
Yes	69	41(59.42)	28(40.58)	0.01
No	41	14(34.15)	27(65.85)	
Multicentricity		
Yes	54	28(51.85)	26(48.15)	0.773
No	56	27(48.21)	28(51.79)	
Tumor size(cm)		
<2	51	27(52.94)	24(47.06)	0.566
≥2	59	28(47.46)	31(52.54)	
Recurrence				
Yes	5	2(40.00)	3(60.00)	1
No	105	53(50.48)	52(49.52)	

In addition, we analyzed the relative expression of *AGAP2-AS1* in PTC cell lines (K1, TPC1, IHH4, and BCPAP) and in normal human thyroid follicular epithelial cells (Nthy-ori 3-1). Compared with Nthy-ori 3-1 cells, the *AGAP2-AS1* expression was increased in TPC1, K1, and IHH4 cells (Figure 1C).

### Knockdown of *AGAP2-AS1* impairs migration and invasion of PTC cells

**Figure 2:**
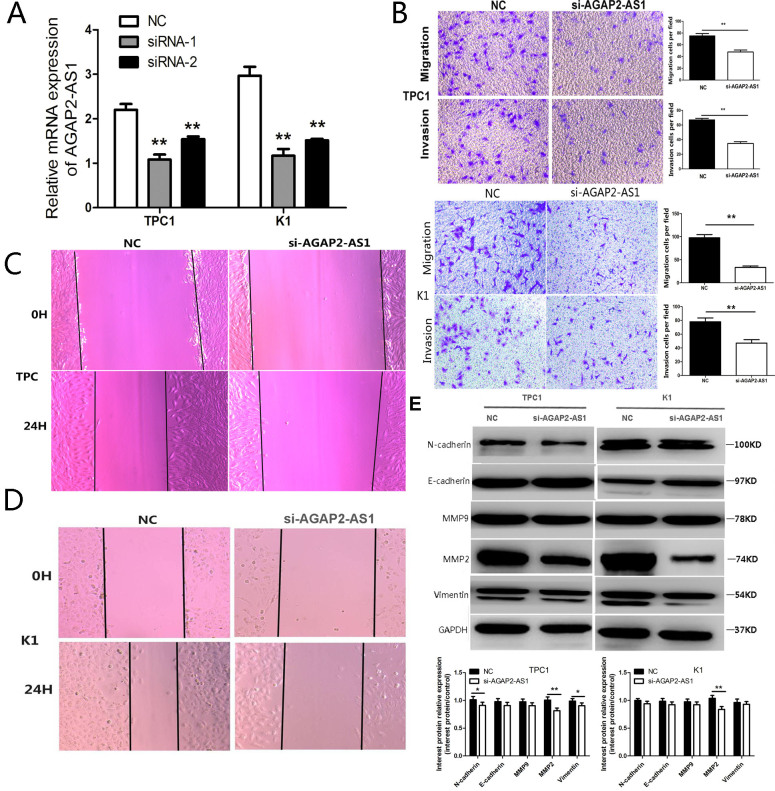
**Knockdown of *AGAP2-AS1* inhibits invasion and migration in PTC cells.** (**A**) RT-PCR of *AGAP2-AS1* after si-RNA suppression in PTC cells; **P < 0.01 *vs*. NC. (**B**) Transwell assay in PTC cells after transfection with si-*AGAP2-AS1* or NC. Data are presented as the mean ± SD, analyzed using independent samples t-test. **P < 0.01 *vs*. NC. (**C**, **D**) Wound healing assay in PTC cells after transfection with *si-AGAP2-AS1* or NC. E. Western analysis of N-cadherin, E-cadherin, MMP2, MMP9, and Vimentin after transfection with si-*AGAP2-AS1* or NC in PTC cells; **P < 0.01, *P < 0.05 *vs*. NC.

To investigate the role of *AGAP2-AS1* in PTC cells, we first used two short interfering RNA (siRNA) sequences to knockdown the expression of *AGAP2-AS1.* As shown in Figure 2A, siRNA1 efficiently suppressed the *AGAP2-AS1* expression in K1 and TPC1 cells; thus, it was used to investigate the function of *AGAP2-AS1* in PTC. Importantly, *AGAP2-AS1* suppression decreased invasion and migration of PTC cells analyzed by Trans-well assay (Figure 2B). In addition, *AGAP2-AS1* knockdown decreased PTC cell proliferation and migration analyzed by wound-healing assay (Figure 2C, 2D). We also analyzed the levels of proteins involved in migration and invasion using western blotting. While there was no significant difference in the levels of E-cadherin, N-cadherin, MMP9, and vimentin between *AGAP2-AS1* knockdown and control cells, *AGAP2-AS1* suppression significantly decreased the level of MMP2 (Figure 2E), indicating that *AGAP2-AS1* promotes migration and invasion of PTC cells by increasing the expression of MMP2.

### *AGAP2-AS1* suppression decreases MMP2 expression by upregulating miRNA-424-5p in PTC cells

**Figure 3:**
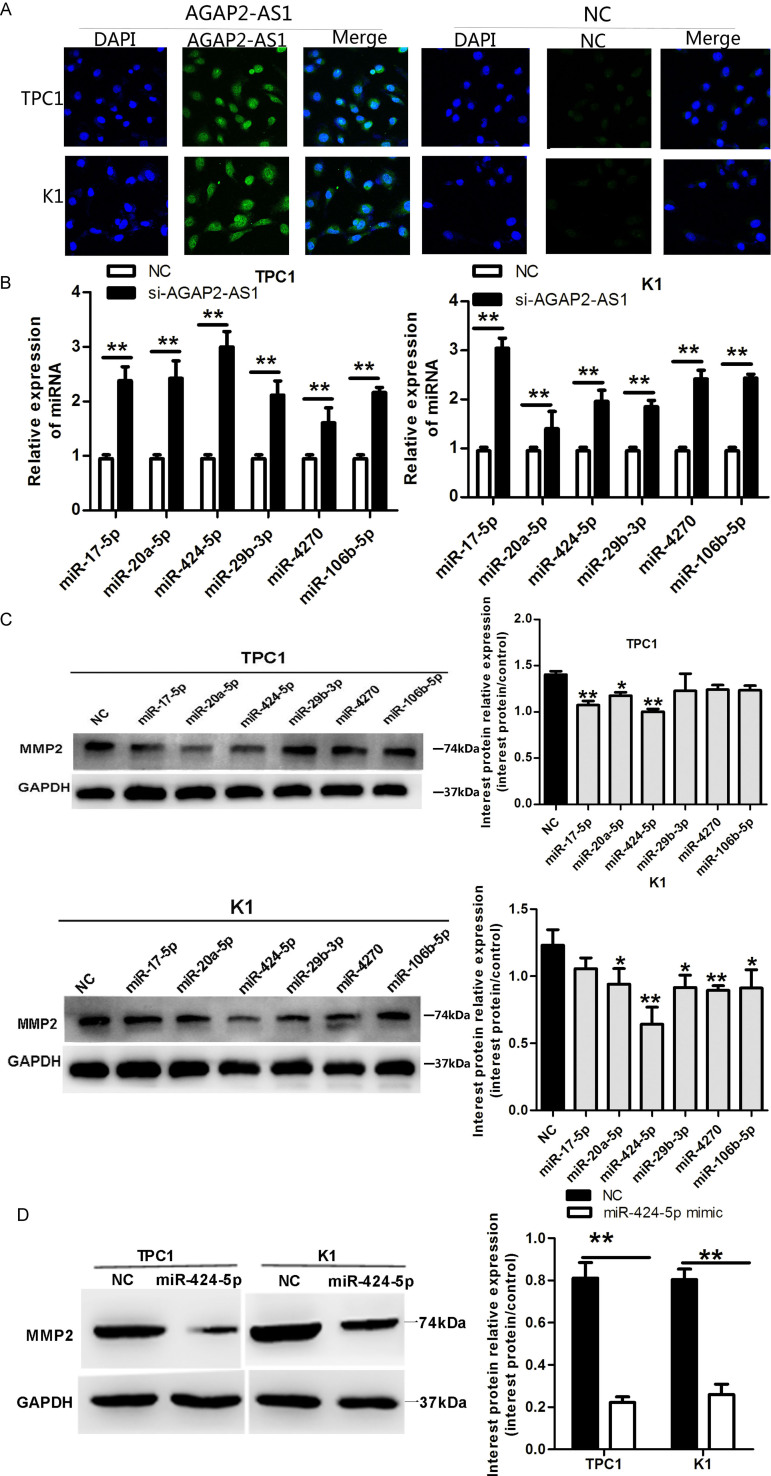
***AGAP2-AS1* suppression decreases MMP2 expression by upregulating miRNA-424-5p in PTC cells.** (**A**) FISH analysis demonstrating cytoplasmic localization of *AGAP2-AS1* in TPC1 and K1 cells. (**B**) qRT-PCR of miRNAs in TPC1 and K1 cells after *AGAP2-AS1* knockdown. Data are presented as the mean ± S.D, analyzed using an independent samples t-test; **P < 0.01 *vs*. NC. (**C**, **D**) Western blotting of MMP2 in TPC1 and K1 cells incubated with miR-424-5p mimic. Data are presented as the mean ± S.D, analyzed using an independent samples t-test; *P < 0.05, **P < 0.01 *vs*. NC.

MiRNAs and lncRNAs interact via miRNA recognition sequences, in which the lncRNA acts as a competing endogenous RNA (ceRNA), resulting in sequestration of the miRNA, and inhibition of the miRNA regulatory effect on the target mRNA [20, 21]. The ceRNA mechanism usually occurs in the cytoplasm; this is consistent with the canonical view that miRNAs act in the cytoplasm [22]. To elucidate the mechanism of how *AGAP2-AS1* upregulates the MMP2 expression, we first analyzed the cellular localization of *AGAP2-AS1* using immunofluorescence. As shown in Figure 3A, *AGAP2-AS1* was localized mainly in the cytoplasm in TPC1 and K1 cells. To investigate whether *AGAP2-AS1* may function as a ceRNA/miRNA sponge, we searched for miRNAs that could bind to both *AGAP2-AS*1 and *MMP2,* using the bioinformatics target prediction tools Targetscan and MirDB. Altogether, 16 miRNAs were identified that might target both *AGAP2-AS*1 and the 3′ UTR of *MMP2*. To test whether expression of these 16 miRNAs is regulated by *AGAP2-AS*1, we analyzed their levels by qRT-PCR after *AGAP2-AS1* knockdown in K1 and TPC1 cells. As shown in Figure 3B, high levels of miR-106b-5p, miR-20a-5p, miR-29b-3p, miR-424-5p, miR-4270, and miR-17-5p were observed in cells with knocked-down *AGAP2-AS1* compared with control cells. To validate these results, we overexpressed these six miRNAs, and analyzed the effect on MMP2 expression. As shown in Figure 3C, 3D, overexpression of miR-424-5p had the most significant effect on the suppression of MMP2 in K1 and TPC1 cells. Together, these data demonstrate that *AGAP2-AS1* is localized mainly in the cytoplasm in PTC cells, and by downregulating the expression of miR-424-5p, it increases the MMP2 expression.

### MiR-424-5p binds to *AGAP2-AS*1 and *MMP2* to suppress invasion and migration of PTC cells

**Figure 4:**
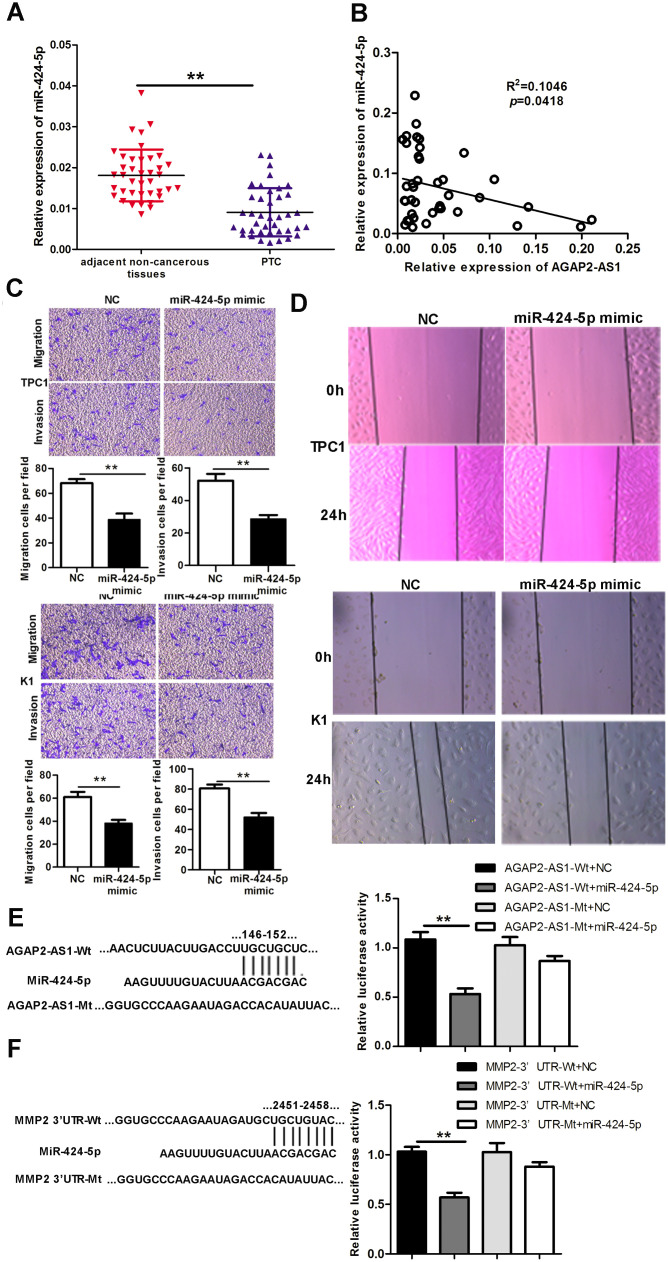
**MiR-424-5p is downregulated in PTC tissues, binds to *AGAP2-AS1* and *MMP2,* and regulates invasion and migration of PTC.** (**A**) Expression of miR-424-5p in 40 pairs of PTC tissues and paired non-cancerous tissues, assessed using qRT-PCR. The Wilcoxon signed-rank test was used to analyze the differences between the two groups; data are presented as the median with a range. **P < 0.01. (**B**) Pearson’s correlation analysis between *AGAP2-AS1* and miR-424-5p expression in PTC tissues (R^2^ = 0.1046, *p* = 0.0418). (**C**) Trans-well assay of invasion and migration of PTC cells after transfection with miR-424-5p mimic or NC. Data are presented as the mean ± S.D, analyzed using an independent samples t-test. **P < 0.01 *vs*. NC. (**D**) Wound healing assay to assess the migration ability of PTC cells after transfection with miR-424-5p mimic or NC. (**E**) Predicted miR-424-5p binding sites in *AGAP2-AS1* (AGAP2-AS1-Wt) and the designed mutant sequence (AGAP2-AS1-Mt) are indicated. HEK 293T cells were transfected with AGAP2-AS1-Wt, AGAP2-AS1-Mt, and the indicated miRNAs, and luciferase reporter assay was conducted. Data are presented as the mean ± S.D, analyzed using an independent samples t-test; **P < 0.01 *vs*. AGAP2-AS1-Wt + NC. (**F**) Predicted miR-424-5p binding sites in the 3′-UTR region of *MMP2* (MMP2-3′UTR-Wt) and the designed mutant sequence (MMP2-3′UTR-Mt) are indicated. HEK 293T cells were transfected with MMP2-3′UTR-Wt or ATAD2-3′UTR-Mt and the indicated miRNAs, and luciferase reporter assay was conducted. Data are presented as the mean ± S.D., analyzed using independent samples t-test. **P < 0.01 *vs*. MMP2-3′UTR-Wt + NC.

Next, we analyzed the miR-424-5p levels in PTC tissues. As shown in Figure 4A, the levels of miR-424-5p were decreased in 40 paired PTC tissues compared with adjacent non-cancerous tissues. Moreover, the levels of *AGAP2-AS1* and *miR-424-5p* negatively correlated in these 40 pairs of PTC tissues (Figure 4B). Overexpression of a miR-424-5p mimic decreased invasion and migration of PTC cells *in vitro*, as analyzed by Trans-well and wound-healing assays (Figure 4C, 4D).

To assess whether miR-424-5p binds to both *AGAP2-AS1* and the 3′ UTR of *MMP2,* we used dual-luciferase reporter assays. Simultaneous transfection of *AGAP2-AS1* wild-type (*AGAP2-AS1* Wt) and the miR-424-5p mimic distinctly decreased the luciferase activity; however, the luciferase activity was not affected when the miR-424-5p mimic and *AGAP2-AS1* mutated-type (AGAP2-AS1 Mt) were cotransfected. The miR-424-5p mimic could also reduce the luciferase activity controlled by wild-type *MMP2*-3′ UTR (MMP2-3′ UTR Wt), but did not show any effect with the mutated-type *MMP2*-3′ UTR in HEK 293T cells (MMP2-3′ UTR Mt). These results indicate that miR-424-5 binds to both *AGAP2-AS1* and *MMP*2, and suggest that *AGAP2-AS*1 upregulates *MMP2* expression by sponging miR-425-5p (Figure 4E, 4F).

### Downregulation of miR-424-5p partly rescues si-AGAP2-AS1-mediated inhibition of invasion and migration in PTC cells

**Figure 5:**
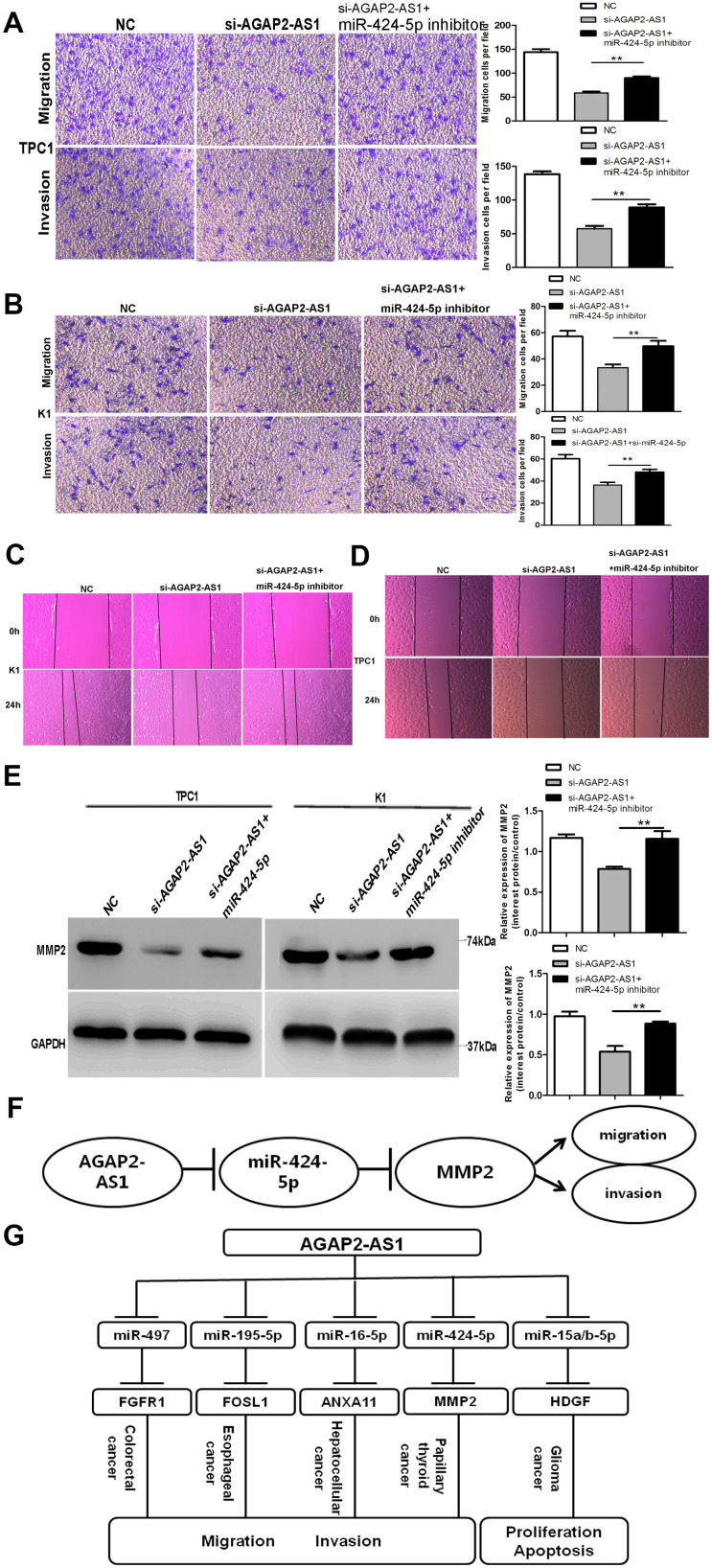
**Downregulation of miR-424-5p partly rescues si-AGAP2-AS1 mediated inhibition of invasion and migration in PTC cells.** (**A**, **B**) Trans-well assays of PTC cell migration and invasion after transfection with si-AGAP2-AS1, or a co-transfection with miR-424-5p inhibitor and si-AGAP2-AS1 or NC. Data are presented as the mean ± S.D., analyzed using an independent samples t-test; **P < 0.01 vs. the si-AGAP2-AS1 group. (**C**, **D**) Wound-healing assay of PTC cell migration and invasion after transfection with si-AGAP2-AS1, or co-transfection with miR-424-5p inhibitor and si-AGAP2-AS1 or NC. (**E**) Western blotting of MMP2 after transfection with si-AGAP2-AS1 or co-transfection with miR-424-5p inhibitor and si-AGAP2-AS1 or NC. Data are presented as the mean ± S.D., analyzed using an independent samples t-test; **P < 0.01 *vs*. the si-AGAP2-AS1 group. (**F**) Schematic illustration of the proposed function of *AGAP2-AS1* in PTC. *AGAP2-AS1* functions as a competing endogenous RNA that upregulates *MMP2* expression and promotes PTC progression by sponging miR-424-5p. (**G**) AGAP2-AS1 played a role as ceRNA in different cancers.

We performed a rescue experiment to validate the interaction between *AGAP2-AS1* and miR-424-5p. When si-AGAP2-AS1 and miR-424-5p inhibitor were expressed simultaneously, the suppressed invasion and migration caused by si-AGAP2-AS were not observed in TPC1 and K1 cells (Figure 5A, 5B). Wound assays showed similar results: Downregulation of miR-424-5p could partly rescue the impact of si-AGAP2-AS1 (Figure 5C, 5D). Western blotting verified the rescue effect of miR-424-5p inhibitor, showing that MMP2 expression was distinctly suppressed when cells were co-transfected with the miR-424-5p inhibitor and si-AGAP2-AS1 compared to cells transfected with si-AGAP2-AS1 alone (Figure 5E). Together, our findings demonstrate that the lncRNA *AGAP2-AS1* upregulates invasion and migration of PTC cells by enhancing the expression of MMP2 by sponging miR-424-5p.

## DISCUSSION

The Human Genome Project provided some tantalizing information regarding the human genome. For example, > 90% of the genome is transcribed; however, only about 2% of the genome is translated [23, 24]. Many of the transcribed sequences and genes have been investigated as diagnostic biomarkers or therapeutic targets [25], including gene mutations, and protein and mRNA markers. Some of them have shown diagnostic value for PTC, including the B-Raf proto-oncogene (BRAF), RAS (GTPases named after rat sarcoma), RET/PTC (rearrangement of the RET proto-oncogene that encodes a receptor tyrosine kinase), PAX8 (Paired Box 8), peroxisome proliferator activated receptor gamma (PPARγ), and galectin-3 [26–28]. In addition, compared with protein-coding genes, many lncRNAs exhibit a tissue-specific expression pattern in multiple human cancers [29].

The lncRNA *AGAP2-AS1* plays a vital role in several types of cancer; it functions mainly as a protein scaffold and ceRNA. *AGAP2-AS1* regulates oncogenesis in non-small-cell lung cancer (NSCLC), in which it is upregulated and binds to *EZH2* (encoding enhancer of zeste homolog 2) and *LSD1* (encoding lysine-specific histone demethylase 1), leading to silencing of *LATS2* (encoding large tumor suppressor kinase 2) and *KLF2* (encoding Kruppel like factor 2), and increased cell proliferation [30]. In addition, *AGAP2-AS1* regulates trastuzumab resistance by targeting *MYD88* (encoding MYD88 innate immune signal transduction adaptor) in breast cancer [31]. *AGAP2-AS1* also regulates gastric cancer development and progression by suppressing p21 and E-cadherin [17]. Furthermore, *AGAP2-AS1* promotes cell proliferation, migration, invasion, and EMT progression in hepatocellular carcinoma via *AGAP2-AS1*/miR-16-5p/ANXA11/AKT axis as ceRNA [16]. Zheng et al. have found that *AGAP2-AS1* regulates glioma cell proliferation and apoptosis by sponging miR-15a/b-5p to upregulate the expression of HDGF [32]. In esophageal cancer, *AGAP2-AS1* suppression increases miR-195-5p expression, and decreases cell proliferation, migration, and invasion [33]. However, the function of *AGAP2-AS1* in PTC was unknown. Our present findings demonstrate that the *AGAP2-AS1* expression is significantly increased in PTC tissues compared with paired noncancerous tissues. The high *AGAP2-AS1* expression in PTC correlates with TNM stage and tumor size. Downregulation of *AGAP2-AS1* in PTC cells inhibits their migration and invasion. Thus, our data show that *AGAP2-AS1* acts as a ceRNA to promote proliferation, migration, and invasion in PTC cells (Figure 5G).

MMPs have a major function in tissue remodeling via enhancing ECM protein turnover, including proteoglycans, elastin, gelatin, collagens and other matrix glycoproteins [34]. MMP-2 is ubiquitously expressed in various cells and tissues, and is involved in many physiological and pathological processes. MMP-2 and its inhibitors TIMP-1 and -2, also function in tumor invasion and metastasis. Indeed, an imbalance between MMP-2 and TIMPs might contribute to tumor progression. Several studies have shown that the expression of MMP2 correlates with lymphatic and vascular invasion, and lymph node metastasis [35, 36]. MMP2 plays a vital role also in thyroid cancer [37, 38]. Our results demonstrate that *AGAP2-AS1* upregulates the *MMP2* expression, thus promoting invasion and migration of PTC cells.

MiRNAs have important roles in cancer, and their ectopic expression can result in cancer development [39]. A recent study has suggested that miR-424-5p functions as a tumor suppressor in breast cancer [40]. MiR-424-5p inhibits *CCNE1* expression (encoding cyclin E1) in ovarian cancer, and inhibits cell cycle by suppressing the E2F transcription factor 1 (E2F1)-RB transcriptional corepressor (PRb) pathway, which promotes apoptosis [41]. Progression of malignant tumors, such as colon carcinoma, epithelial ovarian cancer, and cervical cancer involves miR-424-5p [42, 43]. Our data show that *AGAP2-AS1* is localized predominantly in the cytoplasm in TPC1 and K1 cells. In addition, our findings demonstrate that the expression of miR-424-5p is increased in the *AGAP2-AS1* knockdown PTC cells, and that miR-424-5p downregulates the MMP2 expression. The levels of miR-424-5p inversely correlate with the levels of *AGAP2-AS1* in PTC tissues, and miR-425-5p mimic decreases invasion and migration of PTC cells. Our data suggest that miR-424-5p binds to *AGAP2-AS1* to promote MMP2 expression, resulting in the increased migration and proliferation in PTC cells.

CeRNAs were first described in 2011, and were proposed to form a transcriptome-wide, large-scale regulatory network. As such, they expand the scope of the functional genetic information contained in the human genome. LncRNAs also play important roles in pathological conditions, especially cancer [44]. CeRNAs produce their effects using microRNA response elements (MREs) as the letters of a new language. This type of noncoding transcript includes lncRNAs [45], pseudogenes [46], and circular RNAs [47], which contain binding sequences for microRNAs. The ceRNAs reduce the action of their cognate miRNAs on their mRNA targets by reducing the amount of free microRNAs. Several lncRNAs, acting as ceRNAs, have been reported to play a vital role in cancers, such as small cell lung cancer [48] and pancreatic cancer [49]. The results presented here show that miR-424-5 binds to both *AGAP2-AS1* and the 3′ UTR of *MMP2*. Downregulation of miR-424-5p impairs the effect of si-AGAP2-AS1 on PTC cells in terms of invasion, migration, and MMP2 expression.

In conclusion, our results demonstrate that the lncRNA *AGAP2-AS1* is highly expressed in PTC tissues, and positively correlates with TNM stage and lymph node metastasis in PTC. Knockdown of *AGAP2-AS*1 reduces invasion and migration of PTC cells. These findings indicate that *AGAP2-AS1* functions as a ceRNA that promotes *MMP2* expression in PTC by sponging miR-424-5p, and suggest that *AGAP2-AS1* might serve as a potential diagnostic and therapeutic target for PTC.

## MATERIALS AND METHODS

### Patient samples

Paired samples of PTC and adjacent non-cancerous tissues were collected from patients who received surgery at the First Hospital of China Medical University between 2010 and 2017. The sampled tissues were examined pathologically, frozen in liquid nitrogen, and stored at −80 °C. Their clinicopathological characteristics, such as gender, age, extrathyroidal extension, lymph node metastasis, multicentricity, tumor-node-metastasis (TNM) stage, and tumor size, were recorded.

### RNA extraction and qRT-PCR

RNAiso (Takara, Dalian, China) was used to isolate total RNA. SYBR Premix Ex Taq II (Takara, Shiga, Japan) was used to perform qRT-PCR on a LightCycler 480 system (Roche Molecular Systems Inc., Branchburg, NJ, USA) according to the manufacturers' instructions. The cycling conditions comprised an initial denaturation at 95 °C for 30 s; followed by 40 cycles of denaturation at 95 °C for 5 s, annealing at 60 °C for 30 s; and dissociation at 95 °C for 60 s, 55 °C for 1 min, and 95 °C for 30 s. The primer sequences used were: *AGAP2-AS1* 5′- TACCTTGACCTTGCTGCTCTC-3′ (forward) and 5′-TGTCCCTTAATGACCCCATCC-3′ (reverse), hsa-miR-424-5p 5′- GCCAGCAGCAATTCATGT-3′ (forward) and 5′- TATGGTTTTGACGACTGTGTGAT-3′ (reverse), *GAPDH* 5′-CAGGAGGCATTGCTGATGAT-3′ (forward) and 5′-GAAGGCTGGGGCTCATTT-3′(reverse).

### Cell culture

The European Collection of Authenticated Cell Culture (ECACC, UK) provided the Nthy-ori 3-1 and K1 cell lines. Professor Meiping Shen (Department of General Surgery, The First Affiliated Hospital of Nanjing Medical University, Nanjing, Jiangsu) gifted the TPC1 cell line. DSMZ (Braunschweig, Germany) provided the BCPAP cell line. The Health Science Research Resources Bank (Osaka, Japan) provided the IHH4 cells. TPC1 cells were cultured in Dulbecco’s modified eagle’s medium (DMEM) supplemented with 15% fetal bovine serum (FBS). K1 cells were cultured in DMEM:Ham’s F12:MCDB 105 (2:1:1) supplemented with 2 mM glutamine and 10% FBS. BCPAP and Nthy-ori 3-1 cell lines were maintained in RPMI-1640 medium containing 10% FBS. IHH4 cells were cultured in a 1:1 mixture of RPMI-1640 and DMEM with 10% FBS. Short interfering RNA (siRNA) and negative control (NC) were from Gene Pharma (Suzhou, China). The si-AGAP2-AS1 (sense) sequence was: 5′- GGCACAACGACAAAUGUCUTT-3′. The negative control (NC) sequence was 5′-UUC UCC GAA CGU GUC ACG UTT-3′. Lipofectamine 2000 was from Invitrogen.

### Cell invasion and migration assays

After 24 h transfection, cell concentration was adjusted to 5 × 10^4^ cells/ml with serum-free medium. The upper chamber of Transwell chamber (Costar; 24-well insert, pore size: 8 μ m) was filled with 200 μl of cell suspension, and the lower chamber was filled with 500 μl of medium with 10% FBS. For the invasion assay, polycarbonate filters coated with 50 μl Matrigel (1:9, BD Bioscience) were placed in a Transwell chamber. Cells were incubated for 12 h for the migration assay, and 36 h for the invasion assay. Cells on the bottom surface of the membrane were fixed with 4% paraformaldehyde and stained with 0.5 % crystal violet. The migratory cells were visualized and counted in five random visual fields per insert under an inverted microscope at 100× magnification (Nikon MicrophotFX, Japan). The experiment was repeated three times.

### Wound healing assay

After 24 hours post-transfection, cells were plated in six-well plates at 2 × 10^5^ cells per well. When cells in each well reached 80% confluence, a 200-μl pipette tip was used to scratch the cell surface. PBS was used to wash off free cells, and the wound was photographed; three images were obtained for each wound. The difference in the wound width at 0 and 24 h was used to measure the extent of wound healing. The experiment was repeated three times.

### Western blotting

A Total Protein Extraction Kit (KeyGEN, Nanjing, China) was used to extract proteins from PTC cells. Proteins (20–30 μg) were separated using 10% SDS-PAGE and transferred onto polyvinylidene fluoride membranes (Millipore, Billerica, MA, USA). The membranes were blocked with 5% skim milk for 2 h, and incubated overnight at 4 °C with primary antibodies against MMP2, E-cadherin, MMP9, N-cadherin (1:2000 dilution; Abcam, Cambridge, MA, USA), and control glyceraldehyde-3-phosphate dehydrogenase (GAPDH) (ZSGB-Bio, Beijing, China; 1:1000 dilution). The membranes were then incubated with secondary antibodies (1:10,000 dilution; Cell Signaling Technology, Danvers, MA, USA), followed by chemiluminescence detection (Thermo Fisher Scientific, Waltham, MA, USA). To quantify the intensity of the protein bands, Image J (NIH, Bethesda, MD, USA) was used.

### Fluorescence *in situ* hybridization analysis (FISH)

A PARIS kit (Life Technologies) was used to separate the nuclear and cytosolic fractions, and RNA FISH probes were designed and synthesized. Briefly, cells were fixed with 4% formaldehyde for 15 min and washed with PBS. After pepsin treatment, the fixed cells were dehydrated through an ethanol series. The cells were air-dried and then incubated with the FISH probes (40 nM) in hybridization buffer (Life Technologies). Thereafter, the slides were washed, dehydrated, and mounted using Prolong Gold Antifade Reagent. Nuclei were stained using 2-(4-amidinophenyl)-1H-indole-6-carboxamidine (DAPI), and cells were visualized and photographed under a fluorescence microscope (DMI4000B, Leica, Wetzlar, Germany). The probe sequences are as follows:

1. 5′-CGTCG ACACC CAGGA GATGC GACCA CAGCT CGCCG GGCAC-3′
2. 5′-TGCCG GAGCG CACAG CGAAA GGGTG TGCGC CAACC GGCAC-3′
3. 5′-TTTGA GAATG GAACT GGAAV GACGA GAGTG TCGTA ACGTC -3′

Negative control is blank control.

### Luciferase reporter assay

The fragment from AGAP2-AS1 containing the putative binding sites for miR-424-5p was amplified by PCR, cloned in the firefly luciferase expression vector pMIR-REPORT (Obio Technology, China), and named as AGAP2-AS1-wild-type (AGAP2-AS1-Wt). To mutate the putative binding sites for miR-424-5p in AGAP2-AS1, the sequence of putative binding site was replaced as indicated and was named as AGAP2-AS1-mutated-type (AGAP2-AS1-Mt). HEK 293T cells were seeded into 96-well plates the day before transfection, and transfected with the pMIR-REPORT-AGAP2-AS1-lncRNA-Wt, or pMIR-REPORT-AGAP2-AS1-lncRNA-Mt, together with the Renilla luciferase-expressing vector pRL-TK (Promega, Madison, WI, USA) and miR-424-5p mimic or NC using Lipofectamine 2000 (Invitrogen). Similarly, wild-type MMP2-3′ UTR (MMP2-3′ UTR-Wt) and mutated-type MMP2-3′ UTR (MMP2-3′ UTR-Mt) containing the putative binding site of miR-424-5p were established and cloned into the Firefly luciferase expression vector pMIR-REPORT (Obio Technology, China). HEK 293T cells were seeded into 96-well plates the day before transfection, and transfected with either the pMIR-REPORT-MMP2-3′ UTR-Wt or the pMIR-REPORT-MMP2-3′ UTR-Mt reporter vector, together with the Renilla luciferase-expression vector pRL-TK (Promega) and miR-424-5p mimic or NC using Lipofectamine 2000 (Invitrogen). After 48 h, the cells were harvested, and firefly and Renilla luciferase activities were measured using the dual-luciferase reporter assay system (Promega).

### Statistical analysis

GraphPad Prism 6.0 (GraphPad Software, La Jolla, CA, USA) and SPSS 21.0 software (IBM Corp. Armonk, NY, USA) were used to perform the statistical analyses. The relationship between *AGAP2-AS*1 expression and the clinicopathological characteristics was examined using the chi-squared test. The results were considered statistically significant at a P-value < 0.05.

### Ethics statement

The Ethics Committee of the First Affiliated Hospital of China Medical University, Shenyang, China approved this study. All study participants provided written informed consent.

## References

[1] Lundgren CI, Hall P, Dickman PW, Zedenius J. Clinically significant prognostic factors for differentiated thyroid carcinoma: a population-based, nested case-control study. Cancer. 2006; 106:524–31. 10.1002/cncr.21653 16369995

[2] Chen AY, Jemal A, Ward EM. Increasing incidence of differentiated thyroid cancer in the United States, 1988-2005. Cancer. 2009; 115:3801–07. 10.1002/cncr.24416 19598221

[3] Cooper DS, Doherty GM, Haugen BR, Kloos RT, Lee SL, Mandel SJ, Mazzaferri EL, McIver B, Pacini F, Schlumberger M, Sherman SI, Steward DL, Tuttle RM, and American Thyroid Association (ATA) Guidelines Taskforce on Thyroid Nodules and Differentiated Thyroid Cancer. Revised american thyroid association management guidelines for patients with thyroid nodules and differentiated thyroid cancer. Thyroid. 2009; 19:1167–214. 10.1089/thy.2009.0110 19860577

[4] Grant CS. Recurrence of papillary thyroid cancer after optimized surgery. Gland Surg. 2015; 4:52–62. 10.3978/j.issn.2227-684X.2014.12.06 25713780 PMC4321046

[5] Pacini F, Castagna MG. Approach to and treatment of differentiated thyroid carcinoma. Med Clin North Am. 2012; 96:369–83. 10.1016/j.mcna.2012.01.002 22443981

[6] Fröhlich E, Wahl R. The current role of targeted therapies to induce radioiodine uptake in thyroid cancer. Cancer Treat Rev. 2014; 40:665–74. 10.1016/j.ctrv.2014.01.002 24485648

[7] Landa I, Ibrahimpasic T, Boucai L, Sinha R, Knauf JA, Shah RH, Dogan S, Ricarte-Filho JC, Krishnamoorthy GP, Xu B, Schultz N, Berger MF, Sander C, et al. Genomic and transcriptomic hallmarks of poorly differentiated and anaplastic thyroid cancers. J Clin Invest. 2016; 126:1052–66. 10.1172/JCI85271 26878173 PMC4767360

[8] Xing M. Molecular pathogenesis and mechanisms of thyroid cancer. Nat Rev Cancer. 2013; 13:184–99. 10.1038/nrc3431 23429735 PMC3791171

[9] Rinn JL, Chang HY. Genome regulation by long noncoding RNAs. Annu Rev Biochem. 2012; 81:145–66. 10.1146/annurev-biochem-051410-092902 22663078 PMC3858397

[10] Bartel DP. MicroRNAs: genomics, biogenesis, mechanism, and function. Cell. 2004; 116:281–97. 10.1016/s0092-8674(04)00045-5 14744438

[11] Li L, Chang HY. Physiological roles of long noncoding RNAs: insight from knockout mice. Trends Cell Biol. 2014; 24:594–602. 10.1016/j.tcb.2014.06.003 25022466 PMC4177945

[12] Santosh B, Varshney A, Yadava PK. Non-coding RNAs: biological functions and applications. Cell Biochem Funct. 2015; 33:14–22. 10.1002/cbf.3079 25475931

[13] Huang G, Jiang H, Lin Y, Wu Y, Cai W, Shi B, Luo Y, Jian Z, Zhou X. lncAKHE enhances cell growth and migration in hepatocellular carcinoma via activation of NOTCH2 signaling. Cell Death Dis. 2018; 9:487. 10.1038/s41419-018-0554-5 29706630 PMC5924759

[14] Sun W, Lan X, Zhang H, Wang Z, Dong W, He L, Zhang T, Zhang P, Liu J, Qin Y. NEAT1_2 functions as a competing endogenous RNA to regulate ATAD2 expression by sponging microRNA-106b-5p in papillary thyroid cancer. Cell Death Dis. 2018; 9:380. 10.1038/s41419-018-0418-z 29515109 PMC5841310

[15] Hui B, Ji H, Xu Y, Wang J, Ma Z, Zhang C, Wang K, Zhou Y. RREB1-induced upregulation of the lncRNA AGAP2-AS1 regulates the proliferation and migration of pancreatic cancer partly through suppressing ANKRD1 and ANGPTL4. Cell Death Dis. 2019; 10:207. 10.1038/s41419-019-1384-9 30814490 PMC6393474

[16] Liu Z, Wang Y, Wang L, Yao B, Sun L, Liu R, Chen T, Niu Y, Tu K, Liu Q. Long non-coding RNA AGAP2-AS1, functioning as a competitive endogenous RNA, upregulates ANXA11 expression by sponging miR-16-5p and promotes proliferation and metastasis in hepatocellular carcinoma. J Exp Clin Cancer Res. 2019; 38:194. 10.1186/s13046-019-1188-x 31088485 PMC6518827

[17] Qi F, Liu X, Wu H, Yu X, Wei C, Huang X, Ji G, Nie F, Wang K. Long noncoding AGAP2-AS1 is activated by SP1 and promotes cell proliferation and invasion in gastric cancer. J Hematol Oncol. 2017; 10:48. 10.1186/s13045-017-0420-4 28209205 PMC5314629

[18] Mittal R, Patel AP, Debs LH, Nguyen D, Patel K, Grati M, Mittal J, Yan D, Chapagain P, Liu XZ. Intricate functions of matrix metalloproteinases in physiological and pathological conditions. J Cell Physiol. 2016; 231:2599–621. 10.1002/jcp.25430 27187048

[19] Xiang Y, Li JP, Guo W, Wang DQ, Yao A, Zhang HM, Huang F, Li HH, Dai ZT, Zhang ZJ, Li H, Tan Y, Chen K, et al. Novel interactions between ERα-36 and STAT3 mediate breast cancer cell migration. Cell Commun Signal. 2019; 17:93. 10.1186/s12964-019-0409-4 31409371 PMC6693284

[20] Peng WX, Koirala P, Mo YY. LncRNA-mediated regulation of cell signaling in cancer. Oncogene. 2017; 36:5661–67. 10.1038/onc.2017.184 28604750 PMC6450570

[21] Wilusz JE, Sunwoo H, Spector DL. Long noncoding RNAs: functional surprises from the RNA world. Genes Dev. 2009; 23:1494–504. 10.1101/gad.1800909 19571179 PMC3152381

[22] Huarte M. The emerging role of lncRNAs in cancer. Nat Med. 2015; 21:1253–61. 10.1038/nm.3981 26540387

[23] ENCODE Project Consortium. An integrated encyclopedia of DNA elements in the human genome. Nature. 2012; 489:57–74. 10.1038/nature11247 22955616 PMC3439153

[24] Hangauer MJ, Vaughn IW, McManus MT. Pervasive transcription of the human genome produces thousands of previously unidentified long intergenic noncoding RNAs. PLoS Genet. 2013; 9:e1003569. 10.1371/journal.pgen.1003569 23818866 PMC3688513

[25] Cloutier SC, Wang S, Ma WK, Al Husini N, Dhoondia Z, Ansari A, Pascuzzi PE, Tran EJ. Regulated formation of lncRNA-DNA hybrids enables faster transcriptional induction and environmental adaptation. Mol Cell. 2016; 61:393–404. 10.1016/j.molcel.2015.12.024 26833086 PMC4744127

[26] Hedayati M, Zarif Yeganeh M, Sheikholeslami S, Afsari F. Diversity of mutations in the RET proto-oncogene and its oncogenic mechanism in medullary thyroid cancer. Crit Rev Clin Lab Sci. 2016; 53:217–27. 10.3109/10408363.2015.1129529 26678667

[27] Mottini C, Tomihara H, Carrella D, Lamolinara A, Iezzi M, Huang JK, Amoreo CA, Buglioni S, Manni I, Robinson FS, Minelli R, Kang Y, Fleming JB, et al. Predictive Signatures Inform the Effective Repurposing of Decitabine to Treat KRAS-Dependent Pancreatic Ductal Adenocarcinoma. Cancer Res. 2019; 79:5612–5625. 10.1158/0008-5472.CAN-19-0187 31492820

[28] Murugan AK, Munirajan AK, Alzahrani AS. Long noncoding RNAs: emerging players in thyroid cancer pathogenesis. Endocr Relat Cancer. 2018; 25:R59–82. 10.1530/ERC-17-0188 29146581

[29] Harrow J, Frankish A, Gonzalez JM, Tapanari E, Diekhans M, Kokocinski F, Aken BL, Barrell D, Zadissa A, Searle S, Barnes I, Bignell A, Boychenko V, et al. GENCODE: the reference human genome annotation for the ENCODE project. Genome Res. 2012; 22:1760–74. 10.1101/gr.135350.111 22955987 PMC3431492

[30] Li W, Sun M, Zang C, Ma P, He J, Zhang M, Huang Z, Ding Y, Shu Y. Upregulated long non-coding RNA AGAP2-AS1 represses LATS2 and KLF2 expression through interacting with EZH2 and LSD1 in non-small-cell lung cancer cells. Cell Death Dis. 2016; 7:e2225. 10.1038/cddis.2016.126 27195672 PMC4917662

[31] Dong H, Wang W, Mo S, Chen R, Zou K, Han J, Zhang F, Hu J. SP1-induced lncRNA AGAP2-AS1 expression promotes chemoresistance of breast cancer by epigenetic regulation of MyD88. J Exp Clin Cancer Res. 2018; 37:202. 10.1186/s13046-018-0875-3 30157918 PMC6114182

[32] Zheng Y, Lu S, Xu Y, Zheng J. Long non-coding RNA AGAP2-AS1 promotes the proliferation of glioma cells by sponging miR-15a/b-5p to upregulate the expression of HDGF and activating Wnt/β-catenin signaling pathway. Int J Biol Macromol. 2019; 128:521–30. 10.1016/j.ijbiomac.2019.01.121 30684575

[33] Shen S, Li K, Liu Y, Liu X, Liu B, Ba Y, Xing W. Silencing lncRNA AGAP2-AS1 upregulates miR-195-5p to repress migration and invasion of EC cells via the decrease of FOSL1 expression. Mol Ther Nucleic Acids. 2020; 20:331–44. 10.1016/j.omtn.2019.12.036 32199129 PMC7082499

[34] Cui N, Hu M, Khalil RA. Biochemical and biological attributes of matrix metalloproteinases. Prog Mol Biol Transl Sci. 2017; 147:1–73. 10.1016/bs.pmbts.2017.02.005 28413025 PMC5430303

[35] Augoff K, Grabowski K, Rabczynski J, Kolondra A, Tabola R, Sikorski AF. Expression of decorin in esophageal cancer in relation to the expression of three isoforms of transforming growth factor-beta (TGF-beta1, -beta2, and -beta3) and matrix metalloproteinase-2 activity. Cancer Invest. 2009; 27:443–52. 10.1080/07357900802527221 19212830

[36] Koyama H, Iwata H, Kuwabara Y, Iwase H, Kobayashi S, Fujii Y. Gelatinolytic activity of matrix metalloproteinase-2 and -9 in oesophageal carcinoma; a study using in situ zymography. Eur J Cancer. 2000; 36:2164–70. 10.1016/s0959-8049(00)00297-5 11044656

[37] Guan H, Guo Z, Liang W, Li H, Wei G, Xu L, Xiao H, Li Y. Trop2 enhances invasion of thyroid cancer by inducing MMP2 through ERK and JNK pathways. BMC Cancer. 2017; 17:486. 10.1186/s12885-017-3475-2 28709407 PMC5513028

[38] Huang LL, Wang Z, Cao CJ, Ke ZF, Wang F, Wang R, Luo CQ, Lu X, Wang LT. AEG-1 associates with metastasis in papillary thyroid cancer through upregulation of MMP2/9. Int J Oncol. 2017; 51:812–22. 10.3892/ijo.2017.4074 28731152 PMC5564412

[39] Calin GA, Croce CM. MicroRNA signatures in human cancers. Nat Rev Cancer. 2006; 6:857–66. 10.1038/nrc1997 17060945

[40] Wang J, Wang S, Zhou J, Qian Q. miR-424-5p regulates cell proliferation, migration and invasion by targeting doublecortin-like kinase 1 in basal-like breast cancer. Biomed Pharmacother. 2018; 102:147–52. 10.1016/j.biopha.2018.03.018 29550638

[41] Liu J, Gu Z, Tang Y, Hao J, Zhang C, Yang X. Tumour-suppressive microRNA-424-5p directly targets CCNE1 as potential prognostic markers in epithelial ovarian cancer. Cell Cycle. 2018; 17:309–18. 10.1080/15384101.2017.1407894 29228869 PMC5914728

[42] Xu J, Li Y, Wang F, Wang X, Cheng B, Ye F, Xie X, Zhou C, Lu W. Suppressed miR-424 expression via upregulation of target gene Chk1 contributes to the progression of cervical cancer. Oncogene. 2013; 32:976–87. 10.1038/onc.2012.121 22469983

[43] Zhang J, Liu H, Hou L, Wang G, Zhang R, Huang Y, Chen X, Zhu J. Circular RNA_LARP4 inhibits cell proliferation and invasion of gastric cancer by sponging miR-424-5p and regulating LATS1 expression. Mol Cancer. 2017; 16:151. 10.1186/s12943-017-0719-3 28893265 PMC5594516

[44] Salmena L, Poliseno L, Tay Y, Kats L, Pandolfi PP. A ceRNA hypothesis: the rosetta stone of a hidden RNA language? Cell. 2011; 146:353–58. 10.1016/j.cell.2011.07.014 21802130 PMC3235919

[45] Cesana M, Cacchiarelli D, Legnini I, Santini T, Sthandier O, Chinappi M, Tramontano A, Bozzoni I. A long noncoding RNA controls muscle differentiation by functioning as a competing endogenous RNA. Cell. 2011; 147:358–69. 10.1016/j.cell.2011.09.028 22000014 PMC3234495

[46] Poliseno L, Salmena L, Zhang J, Carver B, Haveman WJ, Pandolfi PP. A coding-independent function of gene and pseudogene mRNAs regulates tumour biology. Nature. 2010; 465:1033–38. 10.1038/nature09144 20577206 PMC3206313

[47] Memczak S, Jens M, Elefsinioti A, Torti F, Krueger J, Rybak A, Maier L, Mackowiak SD, Gregersen LH, Munschauer M, Loewer A, Ziebold U, Landthaler M, et al. Circular RNAs are a large class of animal RNAs with regulatory potency. Nature. 2013; 495:333–38. 10.1038/nature11928 23446348

[48] Zeng F, Wang Q, Wang S, Liang S, Huang W, Guo Y, Peng J, Li M, Zhu W, Guo L. Linc00173 promotes chemoresistance and progression of small cell lung cancer by sponging miR-218 to regulate etk expression. Oncogene. 2020; 39:293–307. 10.1038/s41388-019-0984-2 31477834

[49] Xiong G, Liu C, Yang G, Feng M, Xu J, Zhao F, You L, Zhou L, Zheng L, Hu Y, Wang X, Zhang T, Zhao Y. Long noncoding RNA GSTM3TV2 upregulates LAT2 and OLR1 by competitively sponging let-7 to promote gemcitabine resistance in pancreatic cancer. J Hematol Oncol. 2019; 12:97. 10.1186/s13045-019-0777-7 31514732 PMC6739963

